# SLIT Loss or Sequestration Increases Mammary Alveologenesis and Lactogenesis

**DOI:** 10.17912/micropub.biology.001264

**Published:** 2024-09-06

**Authors:** Oscar Cazares, Min Chen, Julien Menendez, Rut Molinuevo, Gwen Thomas, Jocelyn Cervantes, Michael Yee, Michael Cadell, Megan Durham, Yaqi Zhu, Catherine Strietzel, Jacob W. Bubolz, Lindsay Hinck

**Affiliations:** 1 University of California, Santa Cruz, CA, USA; 2 Zoetis (United States), Kalamazoo, MI, United States

## Abstract

SLITs comprise a family of secreted proteins that function as ligands for Roundabout (ROBO) receptors. Previous research showed that ROBO1 promotes the differentiation of milk-producing alveolar cells by inhibiting Notch signaling in mammary luminal cells. Here, we show enhanced alveolar development and increased milk production in Slit2-/-;Slit3-/- knockout mammary gland epithelia. This result can also be achieved by intraperitoneal delivery of recombinant ROBO1 extracellular domain fragment, ROBO1-5Ig-Fc, which sequesters SLITs. Together, our phenotypic studies suggest that SLITs restrict alveologenesis and lactogenesis by inhibiting ROBO1.

**Figure 1.**
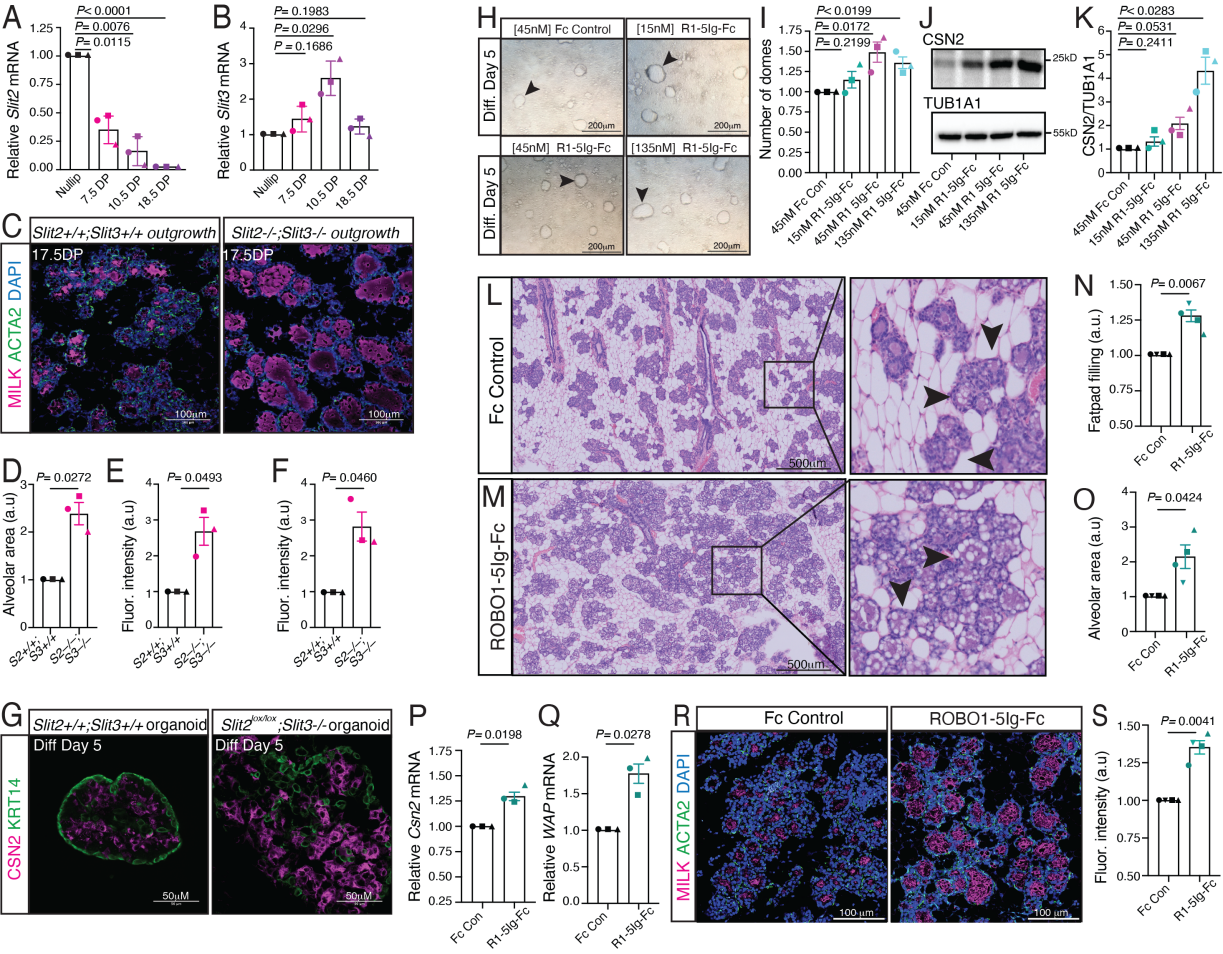
(A, B) Slit2 and Slit3 expression during pregnancy as detected by RT-qPCR (Welch’s t-test). (C-E) Representative immunofluorescence images (C) of 17.5DP Slit2+/+;Slit3+/+ and Slit2-/-;Slit3-/- tissue outgrowths. Quantification (D, E) show alveolar area (D) and milk (magenta; E); basal cells labeled with ACTA2 (green) (Welch’s t-test). (F, G) Quantification (F) and representative immunofluorescence images (G) of CSN2 (magenta) in Slit2+/+;Slit3+/+ and Slit2-/-;Slit3-/- organoids after 5 days of differentiation (paired Welch’s t-test). Basal cells labeled with KRT14 (green). (H, I ) DIC images (H) and quantification (I) of HC11 milk domes after 5 days of differentiation and treatment with control Fc or ROBO1-5Ig-Fc (two-tailed paired t-test). (J, K) Representative immunoblot (J) with quantification (K) of CSN2 in HC11 cells after 5 days of differentiation and treatment with control Fc or ROBO1-5Ig-Fc (Welch’s t-test). (L-O) Representative images of H&E-stained sections from 17.5DP mammary glands after injection with control Fc (L) or ROBO1-5Ig-Fc (M), and quantification of fat pad filling (N) and alveolar area (O) (Welch’s t-test). Insets are magnified images. (P, Q) Csn2 (P) and WAP (Q) expression in 17.5DP mammary glands after injection with control Fc or ROBO1-5Ig-Fc, as detected by RT-qPCR (Welch’s t-test). (R, S) Representative immunofluorescence images (R) and quantification (S) of milk (magenta) in sections from 17.5DP mammary glands after injection with control Fc or ROBO1-5Ig-Fc milk; basal cells labeled with ACTA2 (green) (Welch’s t-test). n=3 independent experiments, 5 images/n D-F, I, K, N, O, S. Data are represented as mean ± SEM.

## Description

During development, the mammary epithelium grows inward from the nipple to generate a tree-like, bi-layered, tubular structure comprising an outer layer of basal myoepithelial cells and an inner layer of luminal epithelial cells that surround a hollow lumen. During pre­gnancy, this epithelial structure grows substantially through progenitor cell expansion and proliferation to generate milk-producing alveoli along its branches (Macias et al., 2012) . We recently showed that the transmembrane ROBO1 receptor is expressed by alveolar myoepithelial cells and functions to enhance milk production by reducing CTNNB1 nuclear translocation and repressing JAG1 expression, thereby curbing Notch activation in neighboring luminal cells (Cazares et al., 2021) . Notch signaling, which stimulates alveolar progenitor cell renewal and expansion, must be suppressed during alveologenesis to promote differentiation of mature, milk-producing alveolar cells (Oakes et al., 2006) . Secreted SLIT proteins (SLITs 1-3) are the ligands for ROBO1, and their function is highly context dependent. For example, SLITs were originally identified as a chemorepellent acting through ROBO1 in the nervous system (Brose et al., 1999) , but there are also examples of them signaling as chemo­attractants through the same receptor (Kellermeyer et al., 2020; Kramer et al., 2001; Ma et al., 2007; Wang et al., 2003) . Here, we explore the function of SLITs during mammary alveologenesis and lactogenesis by examining the consequences of their loss.

In the mammary gland, *Slit2* and *Slit3* are expressed primarily in basal cells while *Slit1* is not detected (Bach et al., 2017; Strickland et al., 2006) . We previously examined the nulliparous phenotype of *Slit2-/-* mammary outgrowths and *Slit3-/-* mammary glands, observing an adhesion phenotype in *Slit2-/-* tissue, but no detectable defects in *Slit3-/-* tissue (Strickland et al., 2006) . To examine *Slit* expression over pregnancy, we used RT-qPCR and observed a steady decline in *Slit2* as pregnancy progressed ( Fig. 1A ). In contrast, *Slit3* increased during early pregnancy reaching its peak at 10.5DP after which it decreased ( Fig. 1B ). To further investigate, we sought to examine the knockout phenotype; however homozygous loss of *Slit2* results in perinatal lethality (Plump et al., 2002) . Consequently, we generated double homozygous *Slit2-/-:Slit3-/-* ( *dKO* ) embryos and followed standard protocols to contralaterally transplant wildtype ( *WT* ) and *dKO* anlage into fat pads of immunocompromised mice that had been precleared of their epithelial tissue (Marlow et al., 2008; Robinson et al., 2000) . We generated tissue for analysis by performing secondary contralateral transplantations using *WT* and *dKO* tissue fragments from the fully developed (8-10 weeks) primary outgrowths produced by anlagen rescue (Marlow et al., 2008; Young, 2000) . We previously showed the efficiency of rescuing *WT* and *dKO* anlage is similar and that *WT* and *dKO* nulliparous secondary outgrowths are also similar in gross morphology (size and branching) (Marlow et al., 2008) . However, the nulliparous secondary *dKO* outgrowths displayed increased proliferation and their ducts contained hyperplastic lesions, suggesting that SLITs play a role in restricting cell growth and governing cellular interactions (Marlow et al., 2008) .

To examine *WT* and *dKO* tissue during pregnancy, the contralaterally-transplanted secondary outgrowths were allowed to fully develop (8-10 weeks) before mating the host animals and harvesting the mammary glands at 17.5DP. By immunostaining tissue sections with anti-milk antibody, we found that *dKO* alveoli were, on average, twice as large as *WT* alveoli and contained more milk ( Fig. 1C -E). SLITs, however, were still present in the stroma in this transplant model (Ahirwar et al., 2021; Chang et al., 2012) . To address this caveat, we obtained a conditional *Slit2* knockout line and generated *Slit2 ^lox/lox^ ;Slit3-/-* animals (Rama et al., 2015, Yuan et al., 2003) . Because tamoxifen administration in these *Slit* *dKO* animals interfered with successful pregnancy, we examined the phenotype by administering tamoxifen to nulliparous animals, collecting the epithelia and generating primary cell organoids (Rubio et al., 2020) . After 5 days of differentiation, immunostaining for CSN2 revealed more milk in *Slit2 ^lox/lox^ ;Slit3-/-* organoids ( Fig. 1F, G ), which also displayed disorganized cells reminiscent of the hyperplastic phenotype previously documented in nulliparous *dKO* mammary outgrowths (Marlow et al., 2008) . Taken together, these data show that loss of *Slit2* and *Slit3* results in enhanced alveologenesis and milk production.

Another way to deplete SLITs is to sequester them by using a ROBO ectodomain (Liu et al., 2004) . We generated and purified ROBO1-5Ig-Fc, comprising the 5 extracellular immunoglobulin (Ig) domains of ROBO1 linked to murine fragment crystallizable region (Fc), and tested this reagent in HC11 cells that undergo a well-established, prolactin-sensitive differentiation process (Cazares et al., 2021; Ball et al., 1988) . We treated competent HC11 cells with ROBO1-5Ig-Fc for 18H, before differentiating the cells for 5 days. We observed an increased number of milk domes with 15nM and 45nM ROBO1-5Ig-Fc treatments ( Fig. 1H, I ). Dome number was not increased with 135nM ROBO1-5Ig-Fc treatment, but the domes appeared larger as reflected by the dose-dependent increase in CSN2 expression, as detected by Western blot ( Fig. 1J, K ). Next, we performed four subcutaneous injections of ROBO1-5Ig-Fc or Fc (7.5mg/kg) into *WT* mice at DP 5.5, 8.5, 11.5, 14.5, and then harvested the mammary glands at DP17.5. Histological analysis of sectioned MGs showed increased epithelial fat pad filling (~35%) and increased alveolar area in ROBO1-5Ig-Fc injected animals ( Fig. 1L -O). This increase in alveolar development was accompanied by increased expression of milk protein genes *Csn2* and *WAP* , as assayed by RT-qPCR ( Fig. 1P, Q ), and increased milk production as assayed by immunostaining ( Figure 1R, S ). These data showed that sequestration of SLITs using ROBO1-5Ig-Fc enhanced alveologenesis, milk protein gene expression and milk production.

Collectively, our data show that knocking out Slits in the murine mammary gland enhances alveologenesis and lactogenesis. Furthermore, we found that depletion of SLITs through injection of ROBO1-5Ig-Fc into WT animals also enhances milk production, an indication that this regulatory axis can be targeted in vivo as a non-hormonal means to increase milk production. Recently, a proteomic analysis of human milk fat globules identified the pathway ­– Regulation of Expression of SLITs and ROBOs – as the top pathway more abundant in milk fat globules compared to milk fat globule membranes, but how this pathway, which contains many proteasomal and ribosomal proteins, affects the function of a milk secreting cell requires further elucidation (Martin Carli et al., 2023) . A limitation of this study is that only the loss-of-function phenotype was examined; further research on the role of SLITs in regulating alveologenesis and milk production will benefit from gain-of-function approaches.

The mammary gland phenotype we identified due to loss of Slits or SLIT sequestration during pregnancy is opposite the one observed in the Robo1-/- mammary gland where decreased alveologenesis and lactogenesis was observed (Cazares et al., 2021) . How SLITs regulate ROBO receptor activity is also a topic of contention. Recent X-ray crystallography studies suggest that a tetrameric assembly involving ROBO1 (cis) dimers interacting back-to-back between adjacent cells (trans) is dissociated and activated by SLIT binding (Aleksandrova et al., 2018) . Another study proposes a different model of trans interaction whereby ROBOs are in an auto-inhibitory assembly that precludes dimerization until SLIT binds and exposes a ROBO dimerization domain (Barak et al., 2019) . Moreover, it is possible there are different mechanisms of SLIT/ROBO signaling when ROBOs face the extracellular matrix where they are free from trans interactions and are regulated instead by interactions with heparin sulfate proteoglycans (Hohenester, 2008) . Taken together, our data provide genetic evidence that during alveologenesis in the mammary gland SLITs either inhibit ROBO1 signaling or potentiate an inhibitory interaction between different ROBOs on adjacent cells (Evans et al., 2015; Kraut et al., 2004) .

## Methods

**Animal Studies:** Slit2-/- were provided by Marc Tessier-Lavigne (Plump et al., 2002) , Slit3-/- mice were provided by David Ornitz (Yuan et al., 2003) , Slit2lox/lox were provided by Alain Chédotal (Rama et al., 2015) . Transplantation studies were performed using Foxn1nu mice (Simonsen Labs). ROBO1-5Ig-Fc studies were performed on timed pregnancy CD-1 female mice (Charles River). All animal procedures were both approved by and conducted in accordance with the guidelines set by the University of California, Santa Cruz (UCSC) Institutional Animal Care and Use Committee (IACUC).

**Tamoxifen administration:** Tamoxifen was dissolved in corn oil in a [20mg/ml] stock solution and given orally [2g/kg body weight] for 3-consecutive days. Slit2lox/lox mice were administered tamoxifen (or corn oil as vehicle control) and experiments were conducted one-month post-administration to allow for maximum depletion of SLIT2.

**Mammary fat pad clearing, and transplantation:** To generate *Slit2-/-;Slit3-/-* embryos, double heterozygote animals were crossed. Mammary anlage from E16-20 *WT* and *Slit2-/-;Slit3-/-* female embryos, generated through independent heterozygous crosses (n=3 independently generated anlage), were rescued and contralaterally transplanted into fat pads of ~6 weeks old, athymic nude ( *Foxn1 ^nu^* ) host females that had been precleared of mammary epithelia at 3 weeks of age (Marlow et al., 2008; Robinson et al., 2000) . To generate additional *WT* and *dKO* tissue for analysis, 8-10 weeks after anlage rescue, similarly sized (~1.5mm ^3^ ) *WT* and *Slit2-/-;Slit3-/-* tissue fragments were dissected from fully developed outgrowths, which had been generated from anlage, and were contralaterally transplanted into ~6 weeks old *Foxn1 ^nu^* hosts that had been precleared at 3 weeks of age (Strickland et al., 2006; Young, 2000) . After 8-10 weeks, animals were mated for timed pregnancies, scored by the presence of a vaginal plug. Plugged mice were considered 0.5 days pregnant (DP) on plug day and embryos were examined at the time of mammary gland harvest to confirm pregnancy timepoint. Contralateral outgrowths were harvested at 17.5 DP.

**ROBO1-Ig5-Fc treatments:** The Expi-CHO Max system was used to prepare large quantities of pure His-ROBO1-5Ig-Fc or His-Fc, followed by purification on a Nickel-NTA column. For in vitro studies, primed HC11 cells were treated with the Fc-Control or ROBO1-5Ig-Fc at the indicated concentrations for 18 hours. ­ For in vivo studies, WT females were subcutaneously injected with either Fc-Control or ROBO1-5Ig-Fc at 7.5mg/kg body weight every 72 hours beginning at 5.5DP (5.5-14.5DP).

**Fat pad filling analysis:** Paraffin-embedded Fc control or ROBO1-5Ig-Fc-injected mammary glands or contralateral outgrowths were sectioned and subjected to H&E staining. Images were analyzed using ImageJ, and percent fat pad filling was calculated by measuring the area occupied by the alveoli.

**3D cell cultures:** Primary cell organoids were grown and processed for high-resolution imaging as previously described (Rubio et al., 2020) . Briefly, primary cells were mixed and grown in Matrigel Growth Factor Reduced (GFR); Phenol Red-Free (Corning, CB-40230C) and cultured in basal medium. After 5 days, growing organoids were washed and cultured in differentiation medium for an additional 5 days. Organoids were liberated from the 3D matrix using ice-cold recovery solution and incubated at 4°C for 60 min. Liberated organoids were fixed with 4% PFA at 4°C for 45 min. Fixed organoids were immunostained using primary antibodies at 4°C for 18 h, washed, and then incubated with secondary antibodies at 4°C for 18 h. Immunostained organoids were mounted with Vectashield® Vibrance™ Mounting Media with DAPI inside three stacked Secure-Seal™ Spacers (Thermo Fisher Scientific, S24735).

**HC11 dome assay:** HC11 cells were obtained from American Type Culture Collection (ATCC) and routinely checked for mycoplasma (Mycoplasma PCR kit, ABM, Cat# G238). Undifferentiated HC11 cells were cultured using in growing medium (RPMI-1640 (Thermo-Fisher, 72400047), supplemented with 10% FBS, [5mg/mL] Insulin (Millipore-Sigma, I6634), [10ng/mL] Epidermal Growth Factor (EGF) (Preprotech, AF-100-15), 1X Anti-Anti at 37°C with 5% CO2. Competent HC11 cells were primed for differentiation by culturing them in priming medium (RPMI-1640 supplemented with, 5% charcoal-stripped-FBS (Equitech Bio, SFBM31), [5mg/mL] Insulin, [1mM] Dexamethasone (Millipore-Sigma, D4902-1G), and 1X Anti-Anti for 18 hours at 37°C with 5% CO2. To induce differentiation, primed HC11 cells were cultured in DIP Medium (RPMI-1640, supplemented with 10% FBS, [5mg/mL] Insulin, [1mM] Dexamethasone (Millipore-Sigma, D4902), 1X anti-anti, and [3mg/mL] Prolactin (NHPP, oPRL-21) at 37°C with 5% CO2.

**Immunofluorescence and microscopy:** Paraffin-embedded tissue was sectioned at a thickness of 5 micrometers and mounted on Superfrost Plus Microscope Slides (Fisher, 12-550-15). Mounted tissue sections were and rehydrated by warming slides to 55°C for 5 minutes, then hydrated by incubating in Xylenes 3 times for 5 minutes, 100% ethanol 2 times for 2 minutes, 95% ethanol for 1 minute, 70% ethanol for 1 minute, 50% ethanol for 1 minute, and diH2O for 10 minutes. Antigen retrieval was performed using antigen unmasking solution (VectorLabs, H3300-250) in a conventional lab microwave. Cells were permeabilized for 20 min in PBS (Thermo-Fisher, 10010023) containing 0.1% Triton-X. Blocking of nonspecific sites was then done using with 10% NDS and 0.1% Triton for 60 min at room temp in a humidifying chamber (VWR, 68432A). For antibodies raised in mouse a M.O.M.® kit was used. Primary and secondary antibodies were diluted and used as described above.

**Western Blotting:­** Whole cell lysates were prepared using 1X NP40 lysis buffer (Themo-Fisher, FNN0021) supplemented with Pierce Protease and Phosphatase inhibitors (Thermo-Fisher, A32959). Cells were washed with ice-cold PBS (Gibco, 14190136), and lysed directly in buffer and kept at 4 °C rocking at 70rpm. Lysed cells were collected and then spun at 14,000 × g at 4 °C for 15 minutes. Equivalent (35-50ug) of each sample was resolved by SDS page and transferred to polyvinylidene difluoride (PVDF) (Millipore-Sigma, IPVH00010), for 60 minutes at 100V. Immunoblots were blocked using 5% (%v/v) fish gelatin for 60 minutes at room temperature. Primary antibodies were incubated overnight at 4°C in a rocker at 65 RPMs. All HRP-conjugated secondary antibodies (Jackson Labs) were used at 1:7500 for 90 minutes at room temp. Immunoblots were developed using Clarity ECL (BioRad), detected using a BioRad Chemi-Doc MP Image, and quantified using ImageLab software (BioRad) as previously described (Le et al., 2016) .

RNA preps and RT-qPCR: Whole-gland total RNA was extracted using Direct-zol RNA MiniPrep Plus (Zymo,R2070). The RNA was further purified with TURBO DNase (Ambion, AM1906) treatment. Total RNA quality was analyzed by agarose gel electrophoresis and quantified with an ND-1000 spectrophotometer (NanoDrop). cDNA was prepared from 500-1000 ng of total RNA using iScript cDNA synthesis kit (BioRad, 1708841). Quantitative RT-qPCR was performed in triplicates using SsoAdvanced Universal SYBR Green Supermix, (Biorad, 1725272). The reactions were run in a BioRad CFX’Connect Real-Time System and CFX Manager software (BioRad) as follows: 95°C for 2 min followed by 40 cycles of 95°C for 15 sec, 60°C for 30 sec and 72°C for 45 sec. The melting curve was graphically analyzed to control for nonspecific amplification reactions. Results were normalized to GAPDH. The primers were all purchased from IDT and the sequences are as follows: mWAP: fwd: 5’-TCTGCCAAACCAACGAGGAGTG bwd: 5’ -AGAAGCCAGCTTTCGGAACACC; mCsn2 fwd: 5’-CCTCTGAGACTGATAGTATT bwd: 5’-TGGATGCTGGAGTGAACTTTA; mSlit2 fwd: 5’-CCCCCTTCACATCAGTTCCC bwd: 5’-TTTCTGCCTATGCGCTGTCA; mSlit3 fwd: 5’-CTAAACCAGACCCTGAACCTGTGGT bwd: 5’ -AAGGTAGAGGGGGCTGTTGCTGCCCACT; mGapdh fwd: 5’ -CATGGCCTTCCGTGTTCCTA bwd: 5’-CCTGCTTCACCACCTTCTTGAT

**Image processing:** Images were processed using Fiji or ZEISS ZEN Imaging Software (Zeiss) and equally adjusted manually if needed. All graphs were generated with GraphPad Prism version 9.0.

**Quantification and statistical analysis:** No statistical method was used to predetermine sample size. Statistical analysis was performed using Prism9 software. Sample size, biological replicates, statistical test, and statistical significance are denoted in the figure legends. P-values higher than 0.05 were considered not statistically significant.

## Reagents

Mouse anti-TUB1A1 clone DM1A (IB, 1:1000), Santa Cruz Biotech; Rabbit anti-KRT14 (IF, 1:1000), Covance; Mouse anti-ACTA2 clone 1A4 (IF, 1:500), Sigma; Rabbit anti-mouse milk (IF, 1:1000) and Rabbit anti-CSN2 monoclonal (IB, 1:4000, IF, 1:500), both kind gifts from Dr. Charles Streuli; Insulin, Millipore-Sigma, I6634; Epidermal Growth Factor (EGF), Preprotech, AF-100-15; Dexamethasone, Millipore-Sigma, D4902); Prolactin (NHPP, oPRL-21); charcoal-stripped-FBS, Equitech Bio, SFBM31.
